# A Switch in Keystone Seed-Dispersing Ant Genera between Two Elevations for a Myrmecochorous Plant, *Acacia terminalis*

**DOI:** 10.1371/journal.pone.0157632

**Published:** 2016-06-16

**Authors:** Fiona J. Thomson, Tony D. Auld, Daniel Ramp, Richard T. Kingsford

**Affiliations:** 1 Landcare Research Manaaki Whenua, Lincoln, New Zealand; 2 Centre for Ecosystem Science, School of Biological, Earth and Environmental Sciences, University of New South Wales, Sydney, New South Wales, Australia; 3 Evolution and Ecology Research Centre, School of Biological, Earth and Environmental Sciences, The University of New South Wales, Sydney, New South Wales, Australia; 4 Office of Environment and Heritage, Sydney, New South Wales, Australia; 5 Centre for Compassionate Conservation, School of Life Sciences, University of Technology Sydney, New South Wales, Australia; Indian Institute of Science, INDIA

## Abstract

The dispersal capacity of plant species that rely on animals to disperse their seeds (biotic dispersal) can alter with changes to the populations of their keystone dispersal vectors. Knowledge on how biotic dispersal systems vary across landscapes allows better understanding of factors driving plant persistence. Myrmecochory, seed dispersal by ants, is a common method of biotic dispersal for many plant species throughout the world. We tested if the seed dispersal system of *Acacia terminalis* (Fabaceae), a known myrmecochore, differed between two elevations in the Greater Blue Mountains World Heritage Area, in southeastern Australia. We compared ant assemblages, seed removal rates of ants and other vertebrates (bird and mammal) and the dominant seed-dispersing ant genera. At low elevations (c. 200 m a.s.l) seed removal was predominantly by ants, however, at high elevation sites (c. 700 m a.s.l) vertebrate seed dispersers or seed predators were present, removing over 60% of seeds from experimental depots when ants were excluded. We found a switch in the keystone seed-dispersing ant genera from *Rhytidoponera* at low elevations sites to *Aphaenogaster* at high elevation sites. This resulted in more seeds being removed faster at low elevation sites compared to high elevation sites, however long-term seed removal rates were equal between elevations. Differences in the keystone seed removalist, and the addition of an alternate dispersal vector or seed predator at high elevations, will result in different dispersal and establishment patterns for *A*. *terminalis* at different elevations. These differences in dispersal concur with other global studies that report myrmecochorous dispersal systems alter with elevation.

## Introduction

Understanding how seed dispersal systems change across landscapes is crucial to understanding plant species’ population dynamics and current and future plant distributions [1]. The dispersal abilities of plant species whose seeds are dispersed by animals (biotic dispersal) can change significantly with variation in the dispersal vector [2]. At the extreme, the disappearance of appropriate seed dispersers can result in dispersal failure for plant species [3]. Accurate knowledge on how biotic dispersal vectors change across landscapes will help conservation managers maintain dispersal synergies, mitigating some of the impacts of habitat fragmentation and climate change [4, 5].

Worldwide, over 11 532 plant species are myrmecochorous, i.e. their seeds are dispersed by ants (c. 4% of angiosperms) [6]. Australia is recognised as a myrmecochory ‘hot spot’ with over 1500 myrmecochorous plant species [7]. Factors that can cause variation in myrmecochorous interactions in Australia include disturbance (e.g. fire), vegetation types, soil nutrient levels, soil moisture and the presence of invasive species [8–14] (. Globally, ant communities and ant seed dispersal systems are known to change with elevation [15–17]. Seed removal rates can increase [18] or decrease with elevation [19], as can the abundance of myrmecochorous ant species [15, 19]. In Australia, studies on seed removal by ants have been primarily conducted at low elevations, i.e. < 500 m a.s.l (e.g. [8, 11, 13, 20–26]), with no studies comparing myrmecochory across elevations. We aimed to determine if seed dispersal systems differed between two elevations (c. 200 m and c. 700 m), within a myrmecochory ‘hotspot’; the Greater Blue Mountains World Heritage Area, New South Wales, Australia, where c. 200 plant species (13%) have seeds adapted for dispersal by ants [27].

Traditionally, myrmecochory was thought to be a diffuse mutualism, where many ant species participated in seed dispersal, also described as a diffuse dispersal model. A change in ant community composition could therefore influence seed removal rates, dispersal distances and predation levels, all of which differ among species of ants [12, 24, 25, 28, 29]. Recent work indicates that a keystone model, where one genus takes the majority of seeds, is more common for myrmecochory. In temperate North America *Aphaenogaster* is the keystone genus [19, 30]. In Australia *Rhytidoponera* has been identified as the dominant seed dispersing ant genus [31, 32]. If keystone genera remain present across the landscape, then changes in ant communities may not affect seed removal rates. For example, the main determinant of reduced seed removal rates in Western Australia was an absence of *Rhytidoponera* species, rather than decreasing latitude [31]. We expect that the overall ant community will change with elevation but the myrmecochorous community will not.

Previously in Australia, seeds adapted for ant dispersal were thought to be predominantly removed by ants [7, 23]. More recent Australian studies have shown that emus, native rats and swamp wallabies also remove myrmecochorous seeds [26, 33]. The type of seed removalist (dispersal vector or seed predator) can influence the spatial arrangement of dispersed seeds. Ants disperse seeds relatively short distances compared with other biotic vectors [34]. Therefore it is important to establish if ants are the sole dispersal vectors because under these conditions dispersal distances would be short, whereas multiple dispersal vectors would result in different seed dispersal patterns.

Here, we present the results of three experiments designed to test if a change in elevation altered seed removal rates, the keystone seed dispersing ant genus, ant community composition, and the primary seed removalist. We predicted that 1) seed removal rates would change with elevation; 2) *Rhytidoponera* species would be the dominant seed dispersing ant genera at both elevations; 3) the ant community but not the myrmecochorous ant community would change with elevation; and 4) dispersal would be predominately by ants at all sites.

## Methods

### Study site

The work was carried out under permission from the New South Wales National Parks and Wildlife Service. No specific permits were required for this work because seeds were not moved between sites and *Acacia terminalis* is not a rare species. In addition National Parks and Wildlife Service staff were directly involved and the Office of Environment and Heritage was an industry partner on the project. Our study was within the Greater Blue Mountains World Heritage Area (GBMWHA; 1.03 million ha), New South Wales, Australia, renowned for its extraordinarily high levels of plant diversity (c. 152 families, 484 genera, 1500 species) [35]. The GBMWHA consists mainly of a dissected sandstone plateau, ranging in elevation from (c.15–1362 m), dominated by schlerophyll forest and heathlands with rainforest gullies [35]. We used *Acacia terminalis* (Salisb.) J.F. Macbr. (Family Fabaceae) for our experiments because it occurs along a large elevation gradient within the GBMWHA, up to 1100m a.s.l. and its seeds are dispersed by ants [8, 23, 25, 36]. *Acacia terminalis* can be a shrub or small tree [37]; it has a mean seed mass of 22.85 mg and mean elaiosome mass of 2.29 mg [38].

Sites selected for experiments satisfied four broad criteria: 1) presence of *A*. *terminalis*; 2) similar recent fire history (not burnt within the last 5 years); 3) similar vegetation communities (a combination of Sydney Sandstone Gully forest and Sydney Sandstone Ridgetop Woodland [39]); and 4) sufficient *A*. *terminalis* seed collected within 100 m of the site to run the experiments (>100 undamaged seeds). High predispersal seed predation levels in 2007 for *A*. *terminalis* plants at >800 m a.s.l. meant that insufficient seed was collected to run experiments. These four criteria were essential, but meant that we were restricted to testing eight sites at each of two elevations; eight low elevation sites between 180–285 m a.s.l (33°47'S, 150°35'E) and eight high elevation sites between 680–790 m a.s.l. (33°48 'S, 150°24'E). Each site was 20 x 20 m, separated from the nearest site by at least 400 m. Treatments within and between sites were assumed to be independent as ants disperse seeds over relatively short distances: 2.24 m ±7.19 m but up to 180 m [28]. The maximum distance between sites, at the low and high elevations, was 7 km and 9 km respectively, while the maximum distance between two sites between elevations was 15 km. However, our experimental design meant we could not categorically rule out that sites within an elevation were spatially autocorrelated.

**Fig 1:**
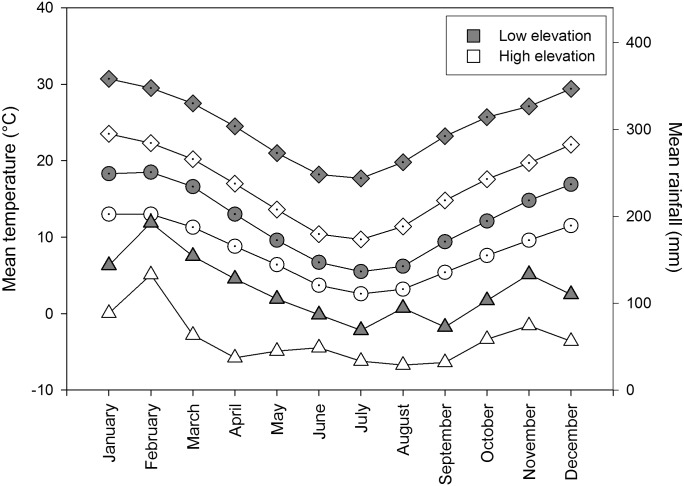
Mean rainfall (triangles) and temperature (diamonds—mean maximum monthly temperature, circles—mean minimum monthly temperature), for high elevation (white) sites (Katoomba; ca. 10km from sites; elevation 1000m; 1907 to 2010) and low elevation (grey) sites (Penrith Lakes; ca. 10km from sites; elevation 25m; 1995 to 2010) from the Australian Bureau of Meteorology.

The high elevation sites were cooler and wetter than the low elevation sites (Fig 1, data from Australian Bureau of Meteorology). Rain can inhibit ant activities so all work was done when it was not raining. Morning and afternoon temperatures were obtained from the nearest weather station to high elevation (Katoomba) and low elevation sites (Penrith Lakes; Australian Bureau of Meteorology). Soil and air temperature are highly correlated in vegetation in this region that have not been recently burnt [40, 41], as with our sites.

To establish which ants removed *A*. *terminalis* seeds and the removal rates of seeds, we observed seed removal from six seed depots randomly placed within each site (n = 16 sites; 96 seed depots). At each depot, five seeds were placed in a circular pattern, >10 cm apart (Total n = 480 seeds). Each seed depot was observed for one hour. At each site, three observation periods were undertaken in the morning and three in the afternoon/evening (n = 96 hours), recording the time a seed was found by an ant and moved by an ant. The seed observation experiment ran in January and February, to coincide with natural seed release in summer. All dispersal distances were recorded, except where heavy litter prevented tracking. Morpho-types of ants observed to interact with seeds were collected after the 1-hr observation period for later identification.

Ant community composition was examined by collecting ants within pitfall traps. Sixteen pitfall traps were placed 5 m apart in a square grid pattern at each site. Traps consisted of plastic tubes (33 mm diameter x 100 mm depth), with a preservative (100% ethanol). Traps were installed at least one month before trapping to avoid disturbance effects. Traps were opened simultaneously at all sites for 96 hours, over two separate periods in March and April 2008. For each site, trap data were pooled for analysis. In the laboratory, ants were identified to genus [42]. Seed-dispersing ant genera were identified from the observation data and [31]. Genera were also assigned functional group categories: Dominant Dolichoderinae, subordinate Camponotini, hot climate specialists, cold climate specialists, tropical climate specialists, cryptic species, opportunists, generalised Myrmicinae and specialist predators (*sensu*[43]). We collected a total of 1,923 ant individuals (low elevation n = 682; high elevation n = 1241), across 27 genera, and seven sub-families. Overall and within elevations the majority of ant individuals found in pitfall traps were mrymecochores (overall = 72%, low elevation 63%, high elevation = 77%).

To establish if ants were the primary seed removalists, we ran an exclusion experiment [26] at 14 sites (seven sites at each elevation). Two sites could not be used because seed loss from rainfall destroyed experimental trials, resulting in insufficient seeds. We had four treatments: only vertebrates (i.e. mammals and birds) excluded; only invertebrates excluded; invertebrates and vertebrates excluded and neither vertebrates nor invertebrates excluded. For each treatment, seeds were placed in a 5-cm diameter petri dish, glued inside a 9-cm diameter petri dish. For treatments that excluded invertebrates, Tanglefoot (an insoluble sticky agent that deters invertebrates from crossing surfaces where it is applied) was smeared between the rims of the inner and outer petri dishes to prevent access to seeds by crawling/walking invertebrates. As mammals and birds readily gained access to the seeds, it was assumed Tanglefoot did not deter vertebrates. For treatments that excluded vertebrates, cages were constructed of a 1 × 1 cm wire mesh and about 15 × 15 cm square and 6 cm high. Each cage was placed over a petri dish and secured with wire stakes. The four treatments were placed together (= one block), with five randomly placed replicates of each block at each site. The number of seeds removed was recorded every 24 hours. The experiment was run simultaneously at all 14 sites during a five-day rain-free period in February 2008.

All data used in analyses are stored at Landcare Research’s datastore: http://dx.doi.org/10.7931/J2F18WNF

### Data Analysis

To compare the effect of elevation on the probability that seeds were found or moved by ants during the observation experiment, we ran two separate binomial logistic regressions [44], using the lmer() function from the lme4 package in R [45]. The response variable for the first logistic regression was proportion of seeds found by ants from a depot. The response variable for the second logistic regression was the proportion of seeds removed by ants from a depot. For both logistic regressions, site was treated as a random factor and elevation as a fixed factor, while temperature at the time of observation was a fixed covariate.

We used contingency tables to investigate the frequency of seed removals between the different ant genera for the high and low elevations. Contingency tables were used as a cautious analytical approach due to the large number of zero’s in the data set, i.e. low numbers of seeds moved by ants during the observation experiment. We chose the three dominant seed removalists (based on the total number of seeds removed), *Rhytidoponera*, *Aphaenogaster* and *Pheidole*. These three genera are known seed dispersers in Australia [43]. Analyses were done in PASW Statistics 18, Release Version 18.0.0 (SPSS, Inc., 2009, Chicago, IL, www.spss.com).

To examine differences in the ant communities between elevations, we constructed Bray-Curtis similarity matrices and conducted analysis of similarity (ANOSIM) with 999 permutations. We also created multidimensional scaling (MDS) plots, using the Bray-curtis similarity matrices to graphically represent the communities. To identify the contribution of individual genera to dissimilarities among the elevations, we calculated similarity percentages. Ant genus abundance data were log transformed before analyses and analyses done in PRIMER 6 [46].

For the exclusion experiment, day 5 seed removal data on whether seeds were removed or not from the depots were analysed using binomial logistic regression [44], using the lmer() function from the LME4 package in R [45]. Day 5 data were used as this was the last day for which all sites were included before rain stopped the experiment. Blocks of the four exclusion treatments were nested within sites, which were nested within elevation. Exclusion treatment and elevation were treated as fixed effects, while blocks and sites were random effects.

## Results

Ants removed 115 of the 480 seeds from depots during the observation experiment. Almost twice as many seeds were removed by ants at low elevation sites (79 out of 240 seeds) than at high elevation sites (36 out of 240 seeds). Seeds found by ants were either found but ignored; found and subsequently moved; or found and the elaiosome removed *in situ*, but the seed not moved. During the observation experiment, 47% of seeds were found by an ant (225 out of 480 seeds), with more seeds found by ants at low elevation (n = 146) than at high elevation sites (n = 79). There was a significant interaction between elevation and temperature at time of observation, influencing whether seeds were found by ants and moved by ants (Table 1). As temperatures increased at high elevation sites more seeds were found by ants and more seeds were removed by ants; this relationship reversed at low elevation sites.

**Table 1:** Results of the two binomial linear regressions for the observation experiment on the proportion of seeds found or moved by ants, across low and high elevations in the Greater Blue Mountains World Heritage Area, including coefficients (±SE), z-value and probability. Temperature is the temperature at time of observation. Significant *P*-values are in bold.

	Seeds Found			Seeds Moved		
Variable	Coefficient ± SE	z value	*P*	Coefficient ± SE	z value	*P*
(Intercept)	-3.02 ± 0.64	-4.717	**< 0.001**	-2.38 ± 0.574	-4.147	**< 0.001**
Low Elevation	3.875 ± 1.01	3.838	**< 0.001**	5.093 ± 1.073	4.747	**< 0.001**
Temperature	0.0785 ± 0.032	2.466	**0.014**	0.100 ± 0.029	3.468	**< 0.001**
Low Elevation * Temperature	-0.146 ± 0.046	-3.162	**0.002**	-0.196 ± 0.047	-4.157	**< 0.001**

**Fig 2:**
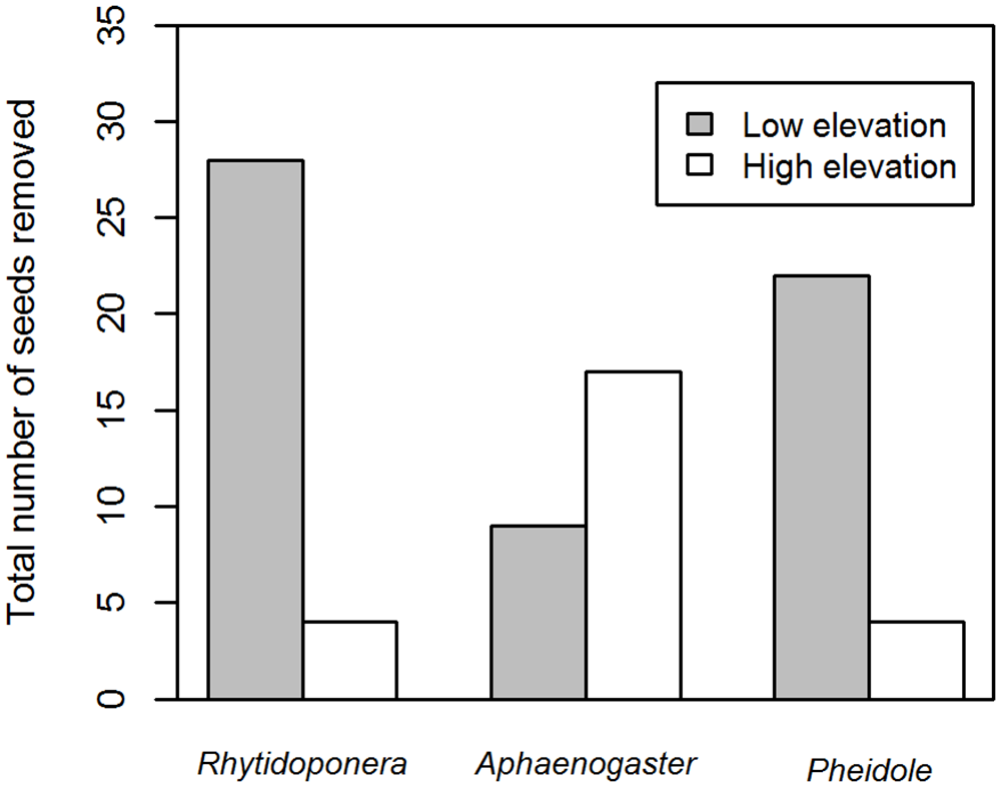
The total number of seeds removed during the observation experiment at low (grey) and high elevation (white) sites by the top three seed-removing ant genera: *Rhytidoponera*, *Aphaenogaster* and *Pheidole*.

Rhytidoponera species were not the dominant seed dispersing ant genera at both low and high elevations sites. There was a significant difference among the three dominant seed removalists (*Rhytidoponera*, *Aphaenogaster* and *Pheidole*) in the number of seeds removed between the two elevations (χ^2^ = 22.92, df = 2, *P* < 0.0001; Fig 2). At low elevation sites, *Rhytidoponera* species were the dominant seed removalists (accounting for 35% of seed removal events; 28 out of 79 events; Fig 2). At high elevation sites, *Aphaenogaster* species were the dominant seed removalists (47% of seed removals; 17 out of 36; Fig 2). Observed *Pheidole* individuals were typically small in body size and poor dispersers, moving seeds short distances (mean = 2.7 cm, maximum = 8 cm, n = 26 observations), or removing the elaiosomes *in situ*. They were more commonly found removing seeds at low elevation sites (Fig 2). Seeds were moved further by *Aphaenogaster* (mean = 11.5 cm, maximum = 80 cm, n = 26 observations) and *Rhytidoponera* (mean = 61.0 cm; maximum = 420 cm, n = 32 observations); a likely underestimate because of our inability to follow ants carrying seeds through leaf litter.

**Fig 3:**
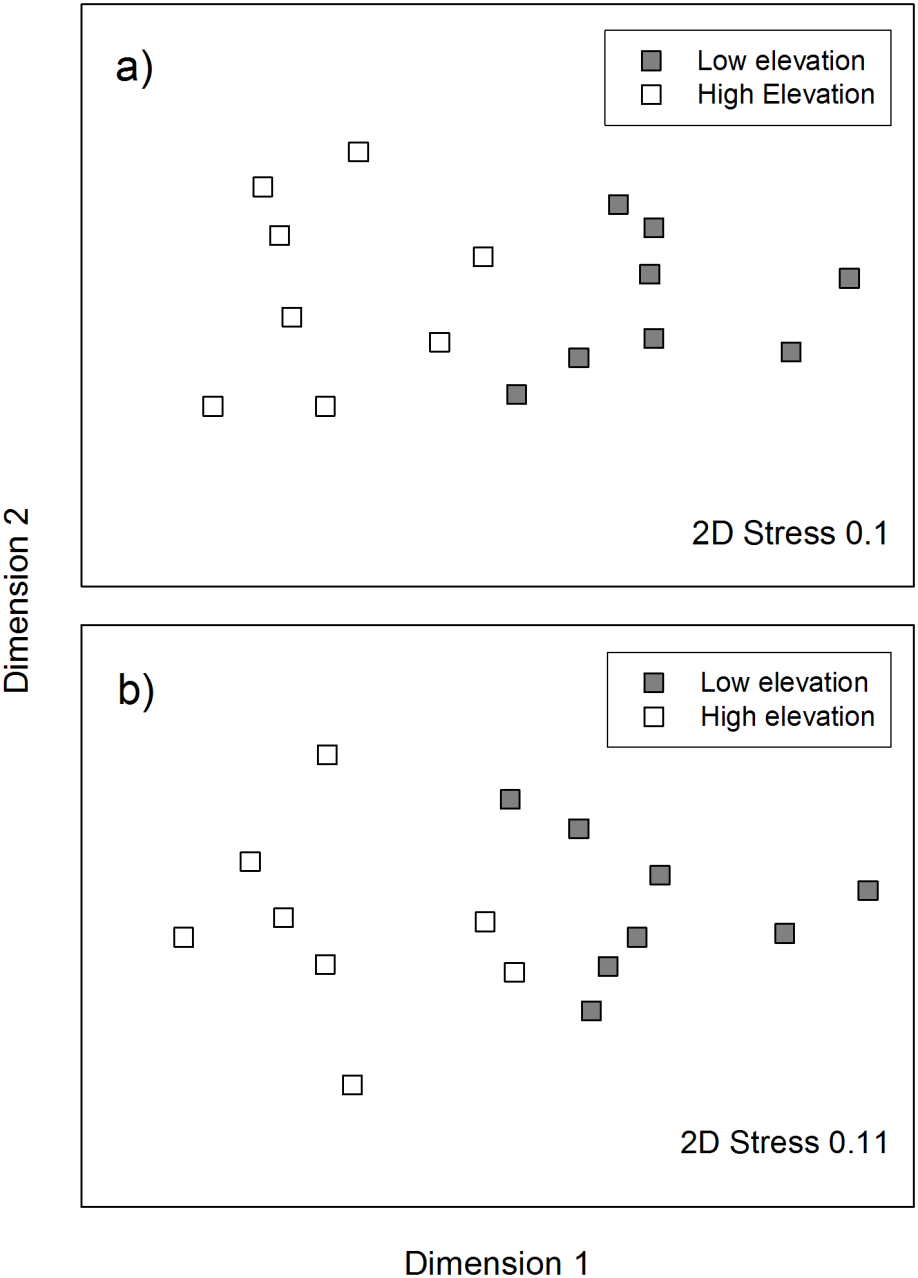
MDS plots of ant communities at low elevation c. 200 m a.s.l. (grey; n = 8) and high elevation sites c. 700 m a.s.l. (white; n = 8) in the Greater Blue Mountains World Heritage Area; a) includes all ant genera, b) includes only the seed-dispersing ant genera.

As predicted, we found significant differences between high and low elevation sites, in the ant community composition (Global R = 0.765; *P* < 0.001; Fig 3a), but we also found significant differences in the seed-dispersing ant community composition (Global R = 0.647; *P* < 0.001; Fig 3b). The major genera contributing to differences in the ant community between elevations were *Anonychomyrma* (contributing 14.4% to the dissimilarity in ant communities between elevations), *Aphaenogaster* (8.53%) and *Rhytidoponera* (8.52%). These genera were also the top contributors to differences in the seed-dispersing ant communities (17.7%, 10.66% and 10.52% respectively). *Anonychomyrma* and *Aphaenogaster* were more abundant at high elevation sites, whereas *Rhytidoponera* was more abundant at low elevation sites (Table 2). Although *Anonychomyrma* is identified as a seed dispersing genus in Australia, it appeared to play a minor role in seed dispersal at our sites despite high abundance. We observed *Anonychomyrma* species removing seeds only four times: three observations at high elevation sites and one at a low elevation site. Seeds moved by *Anonychomyrma* species were taken a maximum of 5 cm before elaiosomes were removed or seeds were abandoned. We found three cold climate specialist genera (*Stigmacros*, *Prolasius*, *Notoncus*). *Stigmacros* and *Prolasius* occurred only at high elevation sites, while *Notoncus* had greater abundance at high elevation than low elevation sites (Table 2). The three hot climate specialist genera occurred only at low elevation sites (*Melophorus*, *Meranoplus*, *Monomorium*; Table 2).

**Table 2:** Mean abundances (± standard error) of ant genera collected from pitfall traps at low and high elevations sites in the Greater Blue Mountains World Heritage Area. Superscript numbers 1, 2, 3 indicate the top three genera that differ between low and high elevation communities. The genera that contain seed-removalists (MYR) are indicated (Y = yes). Superscript letters indicate: a genera that moved seeds, and b genera known as seed predators [31]. The functional group [43] abbreviations are: dominant Dolichoderinae (DD); opportunists (O); subordinate Camponotini (SC); hot & cold climate specialists (HCS & CCS); cryptic species (CS); specialist predators (SP); generalized Myrmicinae (GM).

Family	Genus	Low elevation	High elevation	MYR	Functional Group
Amblyoponinae	*Amblyopone*	-	0.13 ± 0.13	-	-
Dolichoderinae	*Anonychomyrma* ^*1*^	0.13 ± 0.13	54.50 ± 20.70	Y^a^	DD
	*Doleromyrma*	-	4.88 ± 2.52	-	-
	*Iridomyrmex*	8.63 ± 4.04	1.38 ± 0.50	Y	DD
	*Leptomyrmex*	0.88 ± 0.35	3.75 ± 0.94	-	-
Ectatomminae	*Rhytidoponera* ^*2*^	11.75 ± 2.55	2.63 ± 1.38	Y^a^	O
Formicinae	*Camponotus*	1.75 ± 0.37	1.50 ± 0.46	Y	SC
	*Melophorus*	0.25 ± 0.25	-	Y	HCS
	*Notoncus*	10.75 ± 3.78	16.88 ± 5.32	Y^a^	CCS
	*Paratrechina*	4.13 ± 2.02	-	Y^a^	O
	*Plagiolepis*	-	1.38 ± 0.38	-	CS
	*Polyrhachis*	-	0.38 ± 0.38	Y	SC
	*Prolasius*	-	1.63 ± 0.78	Y	CCS
	*Stigmacros*	-	0.25 ± 0.25	-	CCS
Myrmeciinae	*Myrmecia*	-	0.25 ± 0.16	-	SP
Myrmicinae	*Adlerzia*	0.50 ± 0.19	0.38 ± 0.38	-	-
	*Aphaenogaster* ^*3*^	9.50 ± 3.12	38.13 ± 7.52	Y^a^	O
	*Crematogaster*	2.13 ± 0.79	0.50 ± 0.27	Y^a^	GM
	*Epopostruma*	0.13 ± 0.13	-	-	-
	*Meranoplus*	0.63 ± 0.38	-	Y^b^	HCS
	*Monomorium*	3.13 ± 1.09	-	Y^a^	GM/HCS
	*Myrmecina*	-	0.13 ± 0.13	-	-
	*Pheidole*	27.88 ± 5.28	18.5 ± 4.14	Y^a,b^	GM
	*Solenopsis*	1.63 ± 0.56	2.13 ± 0.72	Y	CS
	*Tetramorium*	1.25 ± 0.67	5.88 ± 3.38	Y^a,b^	O
Ponerinae	*Hypoponera*	0.13 ± 0.13	-	-	CS
	*Pachycondyla*	0.13 ± 0.13	-	-	-

**Fig 4:**
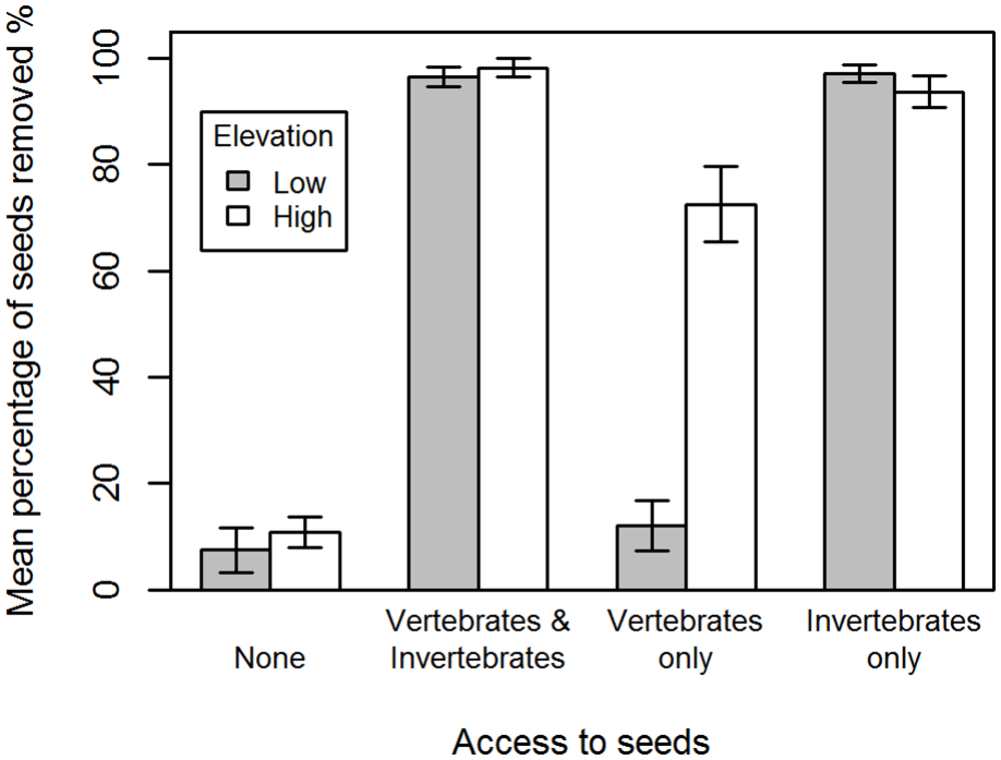
Total mean percentage (± SE) of seeds removed at day five of the exclusion experiment from low elevation sites (grey) and high elevation sites (white). The four exclusion treatments were closed to both invertebrates and vertebrates (None); open to both vertebrates and invertebrates (vertebrates & invertebrates); vertebrates allowed access and invertebrates excluded (vertebrates only); invertebrates allowed access and vertebrates excluded (Invertebrates only).

We found a significant interaction between elevation and invertebrate exclusion, and elevation and vertebrate exclusion (Table 3; Fig 4). At low elevation sites, seed removal was dominated by invertebrates, with exclusion of invertebrates resulting in seed removal events equal to the complete exclusion treatments. At high elevation sites, other seed dispersers or predators removed over 60% of seeds when invertebrates were excluded (Fig 4), resulting in seeds being 80 times more likely to be removed by other seed dispersers or predators at high elevations sites compared to low elevation sites. We recorded seed removals in control treatments (Fig 4), probably due to ‘bridges’ of litter or soil crossing the Tanglefoot, allowing invertebrates access to seeds. At a low elevation site, we observed *Aphaenogaster* individuals carrying soil or leaf litter and placing it on the Tanglefoot creating a ‘bridge’ and then accessing the seeds. We are not aware of any studies reporting similar behaviour in *Aphaenogaster* species. Bridges were removed and Tanglefoot reapplied when seeds remained in the depots.

**Table 3:** Results of the binomial linear regression for the exclusion experiment on seed removal rates, across low and high elevations in the Greater Blue Mountains World Heritage Area, including coefficients (±SE), z-value and probability. The four exclusion treatments for seed depots were: no exclusion (open to both vertebrates and invertebrates); vertebrate access only; invertebrates access only; and closed to both invertebrates and vertebrates. Significant *P*-values are in bold.

Variable	Coefficient ± SE	z value	*P*
(Intercept)	-2.976 ± 0.547	-5.445	**< 0.001**
No Exclusion	9.201 ± 0.882	10.428	**< 0.001**
Vertebrate access only	4.594 ± 0.451	10.193	**< 0.001**
Invertebrate access only	7.473 ± 0.629	11.878	**< 0.001**
Low Elevation	-2.641 ± 0.999	-2.645	0.08
No Exclusion * Low Elevation	2.198 ± 1.485	1.480	0.14
Vertebrate access only * Low Elevation	-3.355 ± 0.774	-4.332	**< 0.001**
Invertebrate access only * Low Elevation	4.158 ± 1.370	3.035	**0.002**

## Discussion

Dispersal systems for a myrmecochorous plant clearly differed between low and high elevations sites; this is the first evidence of this in Australia, and is consistent with other regions of the world [15, 19]. Temperature and rainfall are the most likely reasons for this difference because vegetation types and disturbance history (time since the last fire) were the same at both elevations. The differences may have been driven by spatial separation, although this is unlikely since the distances between elevations (15 km) were similar to distances between sites among elevations (up to 9 km).

We found ant communities and the subset of seed-dispersing ants significantly differed between elevations, as observed elsewhere [15] (Fig 3). Temperature is likely to be a driver of ant community composition: greater abundances of cold climate specialist ant genera were found at high elevation sites compared to low elevation sites and hot climate specialists occurred only at the low elevation sites. The observed patterns in ant community composition were driven by differences in the abundances of three species, two of which (*Rhytidoponera* and *Aphaenogaster*) were observed to remove seeds in this study. Functionally, *Aphaenogaster* and *Rhytidoponera* are classified as opportunistic species; characteristic of disturbed or other habitats supporting low ant diversity [47]. These two genera are good seed dispersers because they are behaviourally subordinate and quickly remove seeds to their nests rather than removing the elaiosome *in situ* to avoid interaction with other ant species [31]. In Australia, only *Rhytidoponera* has previously been identified as a keystone genus for seed dispersal [31], while in temperate North America, *Aphaenogaster* species dominate [29]. We found both these keystone ant dispersers may be important, along with a poor seed dispersing genus (*Pheidole*) at low elevations. Thus the system appears as a combination of the diffuse and keystone models (Fig 2). The relative importance of these keystone genera will also vary depending on local competition for elaiosomes with other ants that do not move seeds, seed dispersers and seed predators.

As predicted, seed removal over the short term was greater at low elevation sites compared to high elevation sites (observation experiment <1 hour). However, temperature at the time of observation may have influenced ant behaviour and therefore seed removal rates. Over a longer term (the exclusion experiment run for 5 days), seed removal rates were slightly higher at low elevation sites, compared to high elevation sites when vertebrates were excluded (Fig 4). This may be explained by *Aphaenogaster* species being more active at night (an effect that would not be detected in the shorter one hr observation experiments) [7, 21], which may lead to higher seed removal rates at night. Most seed removal observation studies in Australia, including this one, have not been conducted during both day and night. This is likely to result in a bias that emphasises the role of diurnal seed dispersing ant taxa, and the importance of *Aphenogaster* species as keystone seed dispersers in Australia may have been underestimated.

In Australia *Aphaenogaster* and *Rhytidoponera* are functionally similar, although their role in the dispersal and establishment success of seeds and seedlings may vary significantly. For example, an Australian experiment found seedlings arising from seeds adapted for dispersal by ants grown in soil from *Aphaenogaster* mounds had greater root and shoot biomass than those grown in soil taken 0.5 m away from nests [48]. Contrastingly, in a *Rhytidoponera*-dominated region in Australia soil nutrient levels around seedlings arising from seeds adapted for ant dispersal, were no greater than the soil nutrient levels around seedlings arising from seeds not adapted for ant dispersal [49]. Mrymecochorous plants at high elevation sites may have better establishment success compared to plants at low elevation sites due to being dispersed by *Aphaenogaster* to nutrient-rich microsites. This could be tested by comparing foliar nutrients and growth rates in seedlings dispersed as seeds by *Aphaenogaster* and *Rhytidoponera* species, while simultaneously testing the soil nutrient properties in which the seedlings exist.

Our evidence suggests *Rhytidoponera* species may disperse seeds greater distances than *Aphaenogaster species*. However, this advantage may be confounded by the greater abundance of *Pheidole* and the presence of *Monomorium* at low elevation sites. *Pheidole* removed more seeds at low elevations, but were poor dispersers, taking seeds short distances, and possibly consuming them [31]. Also, the presence of *Pheidole* and *Monomorium* often prevent long-distance dispersal by other ants, as these genera physically defend seeds and/or remove the elaiosome *in situ*, making them unattractive to other dispersers as we and others have observed [7, 26]. Subsequently a large proportion of the seeds released by plants at low elevations may only be dispersed short distances. The consequences of short dispersal distances include, increased density-dependent mortality of offspring due to competition, pathogen attack and predation [50] and decreased genetic diversity within subpopulations [51].

The predominance of seed dispersal mediated by ants was not consistent across elevations in the Greater Blue Mountains World Heritage Area. The detection of a vertebrate seed-removalist (predator or disperser) at high elevation sites has important implications for understanding plant dispersal curves and recruitment patterns in these plant species. Seeds dispersed through ingestion, attachment or seed-caching have greater mean and maximum dispersal distances than ant-dispersed seeds [34]. In Australia, viable *Acacia blakelyi* seeds have been found in the faeces of kangaroos and emus, implying these vectors can contribute to long distance dispersal events [33]. Our results indicate that *A*. *terminalis* may have considerably different dispersal curves and greater probabilities of long-distance dispersal events at high compared to low elevations. Alternatively, seeds removed by vertebrates may have been consumed. At present we do not know the exact fate of these seeds. Nevertheless, high rates of seed re and as a consequence the ability of *Aphaenogaster* to rapidly move seeds in these habitats may be a key element in reducing seed predation [52]. Regardless of the role of the vertebrate seed removalists, as a seed predator or seed disperser, dispersal probabilities and distances will probably vary between locations. Unravelling these interactions is an excellent opportunity for future work.

Understanding how dispersal systems change across landscapes, including elevation gradients, is critical for accurately predicting distributions of plant species [4, 5]. Our work provides evidence that myrmecochorous dispersal systems change with elevation in Australia, showing a switch in the dominant seed dispersing ants and the addition of vertebrate seed dispersers or seed predators at high elevations that are not present at low elevations. These changes have implications for the establishment success of seedlings, seed dispersal distances and seed predation rates. Our work shows that a change in climate may alter myrmecochorous interactions in Australia through a change in the keystone seed-dispersing ant genera. These considerations are crucial in predicting change associated with global climate change. Future work needs to make comparisons across a greater range of elevations, across different vegetation types and plant species. Futhermore, including manipulative warming experiments (e.g. [53]) would validate patterns seen along elevation gradients as being due to climate rather than other variables such as spatial separation. Another crucial step is to directly connect changes in dispersal systems to changes in plant spatial and population dynamics.

Myrmecochory is an important and common dispersal syndrome throughout Australia. This work in Australia supports previous findings from South Africa and temperate America that myrmecochorous systems change with elevation. Our work also challenges the idea that the genus *Rhytidoponera* is the single keystone seed disperser in Australia and identifies that observation studies on seed removal should be conducted during both night and day to account for seed removal events by nocturnally active ant genera such as *Aphaenogaster*.
